# Predicting potential microbe-disease associations based on auto-encoder and graph convolution network

**DOI:** 10.1186/s12859-023-05611-7

**Published:** 2023-12-14

**Authors:** Shanghui Lu, Yong Liang, Le Li, Rui Miao, Shuilin Liao, Yongfu Zou, Chengjun Yang, Dong Ouyang

**Affiliations:** 1 Faculty of Innovation Enginee, Macau University of Science and Technology, Avenida Wai Long, Taipa, 999078 Macao, Macao Special Administrative Region of China China; 2 School of Mathematics and Physics, Hechi University, No. 42, Longjiang, Hechi, 546300 Guangxi China; 3 Peng Cheng Laboratory, Shenzhen, 518055 Guangdong China; 4 Basic Teaching Department, Zhuhai Campus of Zunyi Medical University, Zhuhai, 519041 Guangdong China; 5 School of Artificial Intelligence and Manufacturing, Hechi University, No. 42, Longjiang, Hechi, 546300 Guangxi China; 6 School of Biomedical Engineering, Guangdong Medical University, No. 1, Xincheng, Zhanjiang, 523808 Guangdong China

**Keywords:** Microbe-disease associations, Auto-enconder, Graph convolution network, Deep forest

## Abstract

The increasing body of research has consistently demonstrated the intricate correlation between the human microbiome and human well-being. Microbes can impact the efficacy and toxicity of drugs through various pathways, as well as influence the occurrence and metastasis of tumors. In clinical practice, it is crucial to elucidate the association between microbes and diseases. Although traditional biological experiments accurately identify this association, they are time-consuming, expensive, and susceptible to experimental conditions. Consequently, conducting extensive biological experiments to screen potential microbe-disease associations becomes challenging. The computational methods can solve the above problems well, but the previous computational methods still have the problems of low utilization of node features and the prediction accuracy needs to be improved. To address this issue, we propose the DAEGCNDF model predicting potential associations between microbes and diseases. Our model calculates four similar features for each microbe and disease. These features are fused to obtain a comprehensive feature matrix representing microbes and diseases. Our model first uses the graph convolutional network module to extract low-rank features with graph information of microbes and diseases, and then uses a deep sparse Auto-Encoder to extract high-rank features of microbe-disease pairs, after which the low-rank and high-rank features are spliced to improve the utilization of node features. Finally, Deep Forest was used for microbe-disease potential relationship prediction. The experimental results show that combining low-rank and high-rank features helps to improve the model performance and Deep Forest has better classification performance than the baseline model.

## Introduction

Microbial communities are collections of microorganisms that live together in the same environment and share a common living space. They are a structural and functional unit that is widely present in ecosystems and can be found in all large organisms and their bodies [1]. Research over the past few decades has shown that microbial communities play a crucial role in human health. During the long process of evolution, microbes form an interdependent and mutually restrictive relationship with the host through individual adaptation and natural selection, while their microenvironment and immune system are in a dynamic equilibrium state [2]. When this dynamic balance is disrupted, the host’s transcription, translation, and DNA repair mechanisms may be affected, which can in turn affect human health. In addition, microbial communities can also play a key role in regulating the efficacy and toxicity of anticancer drugs by regulating host immunity and microbial enzyme degradation mechanisms [3]. For example, changes in the structure of the oral microbiome in a healthy state, that is, changes in the taxonomic composition and relative abundance of the oral microbiome, can lead to the occurrence of dental caries and periodontal disease [4]. Lelouvier, Benjamin, et al. [5] revealed the relationship between changes in the blood microbiome of obese patients and liver fibrosis through qualitative and quantitative analysis of blood bacterial DNA. It has been proven that Helicobacter pylori is associated with a variety of gastrointestinal diseases and was classified as a Group 1 carcinogen by the World Health Organization in 2017 [6–9].

In addition, some microorganisms are considered to be beneficial to human health. Streptococcus thermophilus, which is widely used in the food industry, is considered to be beneficial to human health. The proportion of adults who consume yogurt containing Streptococcus thermophilus while undergoing antibiotic treatment and suffer from antibiotic-associated diarrhea is lower than that of the control group [10]. Bifidobacterium is distributed in both the human oral cavity and vagina, and is abundant in the human digestive tract. Like Streptococcus thermophilus, it is considered beneficial to human health and is widely used in the food and pharmaceutical industries. It is commonly used in the routine treatment of ulcerative colitis and has been proven to have a role in alleviating the disease [11].

As the above research shows, microbial communities can have a crucial impact on human health through a variety of mechanisms. Therefore, identifying potential microbial-disease associations is of great significance for clinical treatment, human health care, drug development, and understanding the relationship between microbes and the human body. In other words, identifying potential microbial-disease associations has practical significance and real-world demand. Further discovery of potential microbial-disease associations not only helps us to better understand the conditions and mechanisms of interaction between microbes and the human body, but also helps to further understand the occurrence and progression mechanisms of microbe-related diseases, and provides new medical solutions for precision treatment, new drug development, and postoperative intervention. However, the number of proven microbial-disease associations is still far from meeting the demand. Therefore, it is necessary and imperative to accelerate the identification of potential microbial-disease associations. Thanks to their efficiency, low cost, and ability to predict potential associations on a large scale of computational models, computational models capable of predicting potential microbial-disease associations have been developed and widely applied. These models can be categorized into four types based on different prediction strategies: matrix decomposition-based methods, label propagation-based methods, path-based methods, and machine learning-based methods.

Although many models for predicting potential microbial-disease associations are based on random walk methods, Qiu et al. [12] have shown that many commonly used random walk methods essentially perform implicit matrix decomposition. Therefore, we combine random walk-based methods with matrix decomposition-based methods for discussion. Matrix decomposition methods refer to representing the target matrix as the result of matrix operations on two or more matrices. Shen et al. [13] proposed a model called CMFHMDA, which is the first microbe-disease association prediction model based on matrix decomposition. CMFHMDA takes the microbe-disease association matrix, microbe Gaussian similarity kernel, and disease Gaussian similarity kernel as inputs to the model and then predicts potential microbe-disease associations. Later, Zou et al. [14] proposed the BiRWHMDA model based on bi-random walk, which constructs a network of microbial similarity and a network of disease similarity through the microbial-disease association matrix, and then connects these two networks to establish a microbial-disease association heterogeneous network and performs bi-random walk on this heterogeneous network to make predictions. Similar models include BiRWMP [15], NMFMDA [16], MSLINE [17], and MVFA [18], etc. The main disadvantage of the matrix decomposition-based methods is that the performance of the model suffers greatly when the matrix is sparse.

The Label Propagation Algorithm (LPA) is a graph-based semi-supervised learning method. The basic idea of LPA is to propagate labels in the data according to pre-given rules. This algorithm was proposed by Zhu et al. [19] in 2002. Since its introduction, the algorithm has been widely used in relation prediction models. For example, Yin et al. [20] and Gao et al [21]. proposed the MDA-MSFLP model and the MKL-LP model, respectively, both of which use the label propagation algorithm to predict potential microbial-disease associations. Zhao et al. [22] proposed a model called PLPMDA, which is based on an improved label propagation algorithm called “Pre-completion-based Label Propagation” to predict potential microbial-drug associations. Similar models include MDLPHMDA [23], NBLPIHMDA [24], etc. The LPA is characterized by its simplicity and efficiency, with the disadvantage of unstable results per iteration and low accuracy.

The basic idea of Path-based methods is to predict the potential relationships by calculating the path score between microbial nodes and disease nodes in a heterogeneous network composed of microbes and diseases. Chen et al [25]. proposed the first model for predicting microbial-disease associations, KATZHMDA, based on the path-based method. This model first calculates the Gaussian interaction profile kernel similarity for microbes and diseases separately, then calculates the KATZ [26] measure and makes predictions. The authors believe that the Gaussian interaction profile kernel similarity and KATZ measure play a crucial role in the performance of KATZHMDA. Inspired by KATZHMDA, Li et al. [27] proposed the BWNMHMDA model, which replaces the KATZ measure with a bidirectional recommendation measure and makes predictions on the resulting bidirectional weighted network. Later, considering the advantages of the KATZ measure and the sparsity of the microbial-disease association matrix, Li et al. [28] proposed the KATZBNRA model based on the Bipartite Network Recommendation Algorithm and KATZ measure to predict potential microbial-disease associations. In addition, there are other models based on the Path-based method, such as PBHMDA [29], WMGHMDA [30], MDPH_HMDA [25], etc. These types of methods are insufficient in extracting high-order structural information from nodes and are also limited by the definition and selection of paths.

Machine learning methods (including deep learning methods) have been widely applied in association prediction in recent years, such as microbe-disease association prediction, microbe-drug association prediction, miRNA-disease association prediction, and recommendation systems. For example, in the prediction of microbe-drug associations, Long et al. [31] utilized GCN (Graph Convolutional Networks) and Conditional Random Field (CRF) to establish a model named GCNMDA for predicting human microbe-drug associations. Subsequently, they proposed the EGATMDA [32] model based on the hierarchical attention mechanism, which demonstrated superior performance in predicting human microbe-drug associations compared to GCNMDA. Sample imbalance is a major issue faced by these types of methods.

In the field of microbe-disease association prediction, Peng et al. [33] proposed ABHMDA, considering the low proportion of positive samples, they used the k-means algorithm to cluster negative samples into 23 categories and randomly selected the same negative samples in each category, then composed these negative samples into negative samples for model training. The ABHMDA model also weights multiple weak classifiers and then forms a strong classifier to predict potential microbe-disease associations. Wang et al. [34] proposed the DSAE_RF model based on the deep sparse autoencoder neural network and random forest. The DSAE_RF model uses a deep sparse autoencoder neural network to extract features of microbe-disease pairs, and then uses the extracted features as inputs to the random forest model to predict potential microbe-disease associations. Inspired by the ABHMDA model, Wang et al. compared the impact of two types of negative sample sampling on model performance, that is, comparing the impact of k-means algorithm sampling and simple random sampling on model performance. The results show that negative sampling through the k-means algorithm can effectively screen reliable negative samples and thereby improve model performance. In addition, graph neural networks have also been well applied in relation prediction. For example, Liu et al. [35] proposed a model based on a multi-component Graph Attention Network (GAT [36]) for microbe-disease association prediction. This model consists of three parts: a decomposer and combiner based on attention mechanism, and a predictor based on a fully connected network. Similarly, Li et al. [37] proposed a model named GATMDA based on GAT for predicting miRNA-disease associations. Wang et al. [38] used Principal Component Analysis (PCA) to extract node features, and then used these features as inputs to a two-layer Relation Graph Convolutional Network (RGCN [39]) to predict potential microbe-disease associations. Jiang et al. [40] proposed a model named KGNMDA, which built a knowledge graph on microorganisms and diseases. KGNMDA used a graph neural network to learn their representations, and proposed a scoring function to predict microbe-disease associations. Models such as MDAGCAN [41], GCNMA [42], MLAGCNMDA [43], etc. also use graph neural network methods.

Although the methods above have achieved certain success in inferring potential microbial-disease associations, these methods also have their own drawbacks. For example, models based on graph neural networks can extract node feature information and topological information well, but in order to prevent “over smoothing”, the number of layers in related models is usually only 2–3 layers, which means that the information obtained by the model is low-order features of the nodes. Although models based on other neural networks can increase the number of layers of the network to a large extent, they cannot handle graph structure data well. Based on this consideration, we propose the DAEGCNDF model. Our model uses a **D**eep Sparse **A**uto-**E**ncoder neural network(**DAE**) to extract deep features of microbial-disease pairs, and uses a **GCN** model to extract low-order features of microbial-disease pairs, then concatenates the deep features with the low-order features and uses Deep Forest for microbial-disease association prediction. The DAE, a model formulated by the combination of stacked and sparse autoencoders and proposed by Lee et al. [44] in 2020, has been widely applied in feature learning and dimension reduction. The Deep Forest(**DF**) model was proposed by Zhou et al. [45] in 2018. This deep model is an extension of the decision tree model, characterized by fewer hyperparameters, determining model complexity by a data-driven approach, and not relying on gradient backpropagation. Experiments show that this model has excellent robustness and performance.

The specific steps can be divided into five. First, we separately calculate the four similarities of microbes and diseases and fuse them. In the second step, the fused similarity matrix is used as the initial input of the GCN module of the model to extract the low-order feature matrix of microbes and diseases. In the third step, a low-order feature vector of microbe-disease pairs is constructed from the extracted low-order feature matrix. In the fourth step, an initial feature vector of microbe-disease pairs is constructed from the fused similarity matrix, and this initial feature vector is input into the DSA module of the model to extract a high-order feature vector of microbe-disease pairs. In the fifth step, the low-order feature vector and high-order feature vector of microbe-disease pairs are concatenated and used for latent microbe-disease association prediction with Deep Forest. Our experimental results show that the model has an average AUC and AUPR of 0.9700 and 0.9690 in 10-fold cross-validation, which fully demonstrates the effectiveness of the model’s predictive performance. In addition, to further evaluate the performance of the model, we also conducted ablation experiments, comparisons of various negative sample selection methods, performance comparisons with other methods, comparisons of various classifiers, and studies on two cases. The experimental results further verify the performance of DAEGCNDF. In summary, our research results will help to further understand the relationship between microbes and diseases, assist in disease diagnosis, treatment and prognosis, and play a supporting role in traditional biological experiments and medical experiments.

Overall, our research has the following main contributions:

1. We use a deep sparse Auto-Encoder neural network to extract high-order feature vectors of microbe-disease pairs.
2. We use GCN to extract low-rank feature matrices of microbes and diseases, and construct low-rank feature vectors of microbe-disease pairs.
3. We concatenate the high-rank feature vectors and low-rank vectors of microbe-disease pairs and use Deep Forest for latent microbe-disease association prediction. The experimental results demonstrate the effectiveness of our model.

## Materials and methods

### Human microbe-disease associations database

Currently, there are three microbial-disease associations datasets, namely HMDAD [46], Disbiome [47], and Peryton [48]. Similar to the research conducted by Wang et al. [34], the data used in this paper is obtained by merging datasets of HMDAD, Disbiome, and Peryton. The basic information of the three datasets above and the integrated dataset used in this paper is shown in Tables 1 and 2, respectively. In this paper, the degree refers to the node degree of the microbe-disease association matrix, that is, the number of edges associated with that node. It should be noted that after merging the three datasets above, we removed duplicate and irrelevant items. As a result, we obtained 1177 microbes, 134 diseases, and 4499 microbe-disease associations, and the microbe-disease associations network was represented by a bipartite graph. An adjacency matrix $\textbf{Y} \in R^{N_m \times N_d}$ was used to represent the microbe-disease associations. In the matrix $\textbf{Y}$, the rows represent $N_m$ microbes, and the columns represent 134 diseases. If a microbe ${m}_{i}$($1 \le i \le N_m$) is associated with a disease ${d}_{j}$(${1 \le j \le N_d}$), then $\textbf{Y}_{ij}=1$, otherwise $\textbf{Y}_{ij}=0$. When $\textbf{Y}_{ij}=1$, we consider it as a positive sample, otherwise, it is considered as a negative sample. In this way, we obtained 4499 positive samples from the integrated dataset(MDAID).

**Table 1:** The basic information about HMDAD, Disbiome, and Peryton

Database	Microbes	Diseases	Associations
HMDAD	292	39	450
Disbiome	1582	352	8645
Peryton	1396	43	4172

**Table 2:** The basic information about the integrated dataset(MDAID)

	Name	Number
Min degree	Microbes	1
	Diseases	1
Max degree	Microbes	59
	Diseases	255
Average degree	Microbes	3.8
	Diseases	33.6
Total	Microbes	1177
	Diseases	134
	Associations	4499

### Diseases similarity

In this study, we employ four distinct methods to calculate disease similarity: semantic similarity, Gaussian Interaction Profile kernel similarity(GIP), cosine similarity, and sigmoid kernel function similarity.

#### Diseases semantic similarity

The calculation of disease similarity is very important for downstream tasks. Xuan [49] proposed a method for calculating similarity based on disease ontology information. The disease similarity calculated by this method is called disease semantic similarity. Since its proposal, disease semantic similarity has been widely used in various researches. Disease ontology information can be obtained from the Human Disease Ontology (DO) [50] ( http://www.disease-ontology.org) or the the Medical Subject Headings (MeSH) database ( https://www.ncbi.nlm.nih.gov/), and each disease in the two database above can be represented as a Directed Acyclic Graph (DAG). Our calculation of disease semantic similarity is based on DAG, and the specific steps are as follows: Firstly, let $DAG({d}_{i}) = ({d}_{i},T({d}_{i}),E({d}_{i}))$ represent the directed acyclic graph of disease ${d}_{i}$, which encompasses disease ${d}_{i}$, its ancestor nodes $T({d}_{i})$, and the set of edges $E({d}_{i})$ that directly connect from the ancestral nodes to node $T({d}_{i})$. The semantic contribution value of disease ${d}_{k}$ to ${d}_{i}$ can then be calculated by using the equation:

1 $$\begin{aligned} SC_{d_{i}} (d_{k}) = {\left\{ \begin{array}{ll} 1,&{} \text {if } d_{k} = d_{i}\\ max\{FC \times SC_{d_{i}} ({d_{k^{'}}})\}, &{} \text {other}\\ \end{array}\right. } \end{aligned}$$

In this context, $d_{k^{'}}$ denotes the children node of $d_{k}$, and *FC* signifies the contributing factor of semantic decay. As per the study by Xuan et al. [49], we set $FC=0.5$. We have determined the contributing factor of disease $d_{i}$ to itself to be 1. Drawing from Eq (1), it can be deduced that an increase in the distance from disease $d_{k}$ to disease $d_{i}$ results in a decrease in the semantic contribution factor. Conversely, a decrease in this distance leads to an increase in the semantic contribution factor. The final semantic value of disease $d_{i}$ can be calculated by using the formula:

2 $$\begin{aligned} SemV(d_{i}) = \sum _{d_{k} \in T(d_{i})}SC_{d_{i}} ({d_{k}}). \end{aligned}$$

The proposition is that diseases with a higher number of shared DAGs are deemed more similar. Based on this premise, the disease semantic similarity between disease $d_{i}$ and $d_{j}$ can be determined by employing the equation:

3 $$\begin{aligned} \textbf{DS}(d_{i},d_{j}) = \frac{\sum \limits _{d_{k} \in T(d_{i}) \cap T(d_{j})}(SC_{d_{i}} (d_{k})+SC_{d_{j}} (d_{k}))}{SemV(d_{i})+SemV(d_{j})}. \end{aligned}$$

#### Gaussian interaction profile kernel similarity for diseases

Due to the excellent performance capabilities of GIP, it has been used in many studies to describe the similarity complement of microbes and diseases. Specifically, the Gaussian interaction profile kernel similarity for any two diseases, denoted as $d_{i}$ and $d_{j}$, can be determined by using the equation:

4 $$\begin{aligned} \textbf{GDS}(d_{i}, d_{j})= & {} exp\left( -\gamma _{d} \Vert \textbf{DB}(d_{i})-\textbf{DB}(d_{j}) \Vert ^2 \right) , \end{aligned}$$

5 $$\begin{aligned} \gamma _{d}= & {} \alpha _{d}/\left( \frac{1}{N_{d}}\sum _{i=1}^{N_{d}} \Vert \textbf{DB}(d_{i}) \Vert ^2 \right) . \end{aligned}$$

In this context, the binary vector $\textbf{DB}(d_{i})$ is equivalent to the *ith* row of the matrix $\textbf{Y}$, which signifies the relationships between disease $d_{i}$ and all microbes. The term $N_{d}=134$ indicates the number of diseases. The value of $\alpha _{d}$ was set to 1, as suggested in the studies by Chen et al.  [51].

#### Cosine similarity for diseases

Cosine similarity is used to evaluate the similarity between two vectors by calculating the cosine of the angle between them. It has been widely applied in various research fields and has demonstrated excellent performance [46, 52]. Therefore, this paper also uses cosine similarity to calculate the similarity between diseases. In particular, the cosine similarity between any two diseases, $d_{i}$ and $d_{j}$, can be determined by employing the subsequent equation:

6 $$\begin{aligned} \textbf{CDS}(d_{i},d_{j})=\frac{\textbf{DB}(d_{i})\cdot \textbf{DB}(d_{j})}{\Vert \textbf{DB}(d_{i}) \Vert \times \Vert \textbf{DB}(d_{j}) \Vert }. \end{aligned}$$

#### Sigmoid kernel function similarity for diseases

Studies have demonstrated that the sigmoid kernel function falls under the category of global kernel functions, thereby enabling the effective extraction of global characteristics from samples. The similarity measure derived from the sigmoid kernel function has found application in the research conducted by Han et al. [53] and Wang et al. [34]. Inspired by their work, this paper also employs the sigmoid kernel function similarity measure to ascertain the similarity between diseases and microbes. For any given pair of diseases, $d_{i}$ and $d_{j}$, their similarity based on the sigmoid kernel function can be computed as follows:

7 $$\begin{aligned} \textbf{SDS}(d_{i},d_{j})=tanh\left( \frac{1}{134}\textbf{DB}(d_{i})\cdot \textbf{DB}(d_{j})\right) . \end{aligned}$$

### Microbes similarity

This section presents four distinct computational techniques for determining microbe similarity, namely functional similarity, Gaussian interaction profile kernel similarity, cosine similarity, and sigmoid kernel function similarity.

#### Microbes functional similarity

The computation of microbial functional similarity hinges on the premise that microbes with similar functions have a higher likelihood of being linked to analogous diseases. Following the same method as Liu et al. [54], we assume that any two microbes $m_{i}$ and $m_{j}$ are associated with disease groups $D_{i}=\{d_{ik}|1 \le k \le p\}$ and $D_{j}=\{d_{jl}|1 \le l \le q\}$ respectively, and the similarity of $d_{ik}$ with disease group $D_{j}$ can be calculated by the following formula:

8 $$\begin{aligned} Sim(d_{ik},D_{j}) = \mathop {max}\limits _{d_{jl} \in D_{j}}\left( \textbf{DS}(d_{ik},d_{jl}) \right) . \end{aligned}$$

Where a is the semantic similarity between disease $d_{ik}$ and $d_{jl}$, which is the element of the disease semantic similarity matrix $\textbf{DS}$ in the $ik-th$ row and $jl-th$ column. Subsequently, the functional similarity between microbes $m_{i}$ and $m_{j}$ can be determined as:

9 $$\begin{aligned} \textbf{FMS}(m_{i},m_{j})= & {} \frac{\sum \limits _{1 \le k \le p}Sim(d_{ik},D_{j})}{p+q}\nonumber \\{} & {} +\frac{\sum \limits _{1 \le l \le q}Sim(d_{jl},D_{i})}{p+q}. \end{aligned}$$

#### Gaussian interaction profile kernel similarity for microbes

In a manner akin to the previously described method for calculating microbe similarities, the GIP similarity between two microbes, denoted as $d_{i}$ and $d_{j}$, can be determined as follows:

10 $$\begin{aligned} \textbf{GMS}(m_{i}, m_{j})= & {} exp\left( -\gamma _{m} \Vert \textbf{MB}(m_{i})-\textbf{MB}(m_{j}) \Vert ^2 \right) , \end{aligned}$$

11 $$\begin{aligned} \gamma _{m}= & {} \alpha _{m}/\left( \frac{1}{N_{m}}\sum _{i=1}^{N_{m}} \Vert \textbf{MB}(m_{i}) \Vert ^2 \right) . \end{aligned}$$

Within this framework, the binary vector $\textbf{MB}(m_{i})$ aligns with the *ith* column of matrix $\textbf{Y}$, thereby delineating the associations between microbe $m_{i}$ and all encompassing diseases. In a similar vein, the value of $\alpha _{m}$ is designated as 1.

#### Cosine similarity for microbes

In a manner akin to the computation of cosine similarity between two diseases, the cosine similarity between two microbes can be ascertained utilizing the subsequent equation:

12 $$\begin{aligned} \textbf{CMS}(d_{i},d_{j})=\frac{\textbf{MB}(m_{i})\cdot \textbf{MB}(m_{j})}{\Vert \textbf{MB}(m_{i}) \Vert \times \Vert \textbf{MB}(m_{j}) \Vert }. \end{aligned}$$

#### Sigmoid kernel function similarity for microbes

Similarly, the sigmoid kernel function similarity between microbes can be computed in the following equation:

13 $$\begin{aligned} \textbf{SMS}(m_{i},m_{j})=tanh\left( \frac{1}{1177}\textbf{MB}(m_{i})\cdot \textbf{MB}(m_{j})\right) . \end{aligned}$$

### Multi-source features fusion for microbes and diseases

The fusion of multi-source features has been proven by many studies to be beneficial in improving model performance. Therefore, we fuse the four disease features and four microbial features above. The fusion operations are performed using Eqs. (14) and (15) respectively to obtain the fused disease and microbial features.

14 $$\begin{aligned} \textbf{FuD}(d_{i},d_{j})= & {} \frac{\textbf{DS}+\textbf{GDS}+\textbf{CDS}+\textbf{SDS}}{4}. \end{aligned}$$

15 $$\begin{aligned} \textbf{FuM}(m_{i},m_{j})= & {} \frac{\textbf{FMS}+\textbf{GMS}+\textbf{CMS}+\textbf{SMS}}{4}. \end{aligned}$$

### Negative sample selection method

In this study, due to the fact that negative samples far outnumber positive samples, balancing positive and negative samples and selecting high-quality negative samples for model training can improve model performance, thereby enhancing the efficiency and effectiveness of the model in predicting potential microbe-disease associations. Peng et al. [33] and Wang et al. [34], in their research, used the KMeans algorithm to cluster negative samples into 23 classes. They then randomly selected an equal number of samples from each cluster as negative samples. Finally, they combined the selected negative samples with all positive samples to serve as training samples for the model. In their research, the parameter *k* of the KMeans algorithm was set to 23. Their experiments showed that selecting negative samples through the KMeans algorithm can improve the model’s AUC and AUPR by about 2$\%$. Inspired by their work, we used four clustering algorithms for negative sample selection: KMeans, Gaussian mixture, Spectral coclustering, and Spectral biclustering. We also conducted an evaluation of these four negative sampling methods. Like the aforementioned research, we retained all positive samples. When conducting experiments on the MDAID dataset, we selected 4508 negative samples, while for the HMDAD dataset, we selected 450 negative samples.

**Fig. 1:**
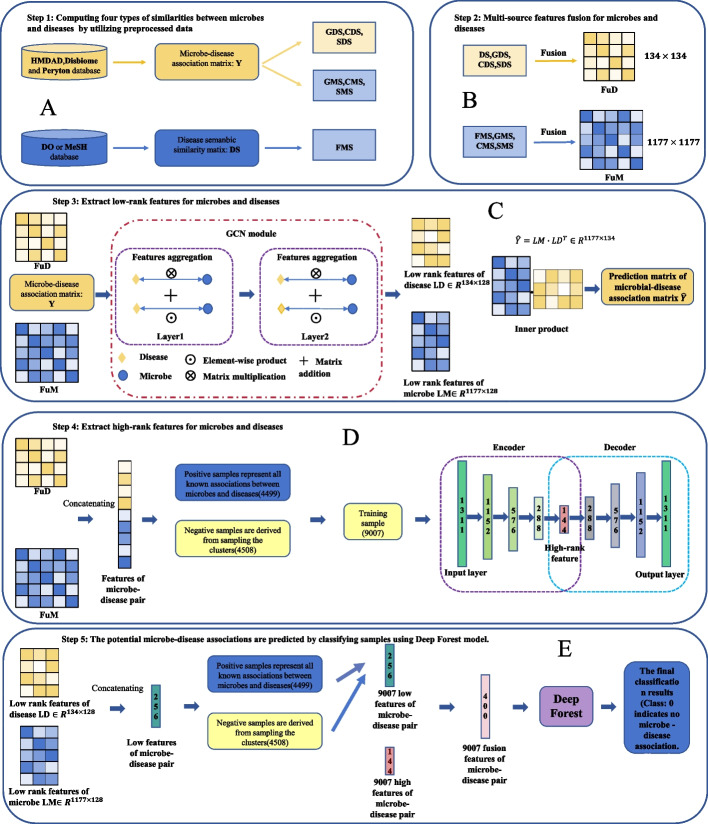
The overview of DAEGCNDF framework. **A** Similarity calculation. **B** Similarity fusion. **C** Extraction of low-rank features. **D** Extraction of high-rank features. **E** Feature fusion and prediction using Deep Forest model

### Model framework

Deep Auto-Encoder models have good representational efficiency and can extract rich data features. The work of Wang et al. [34] also shows that the classification effect extracted based on the deep Auto-Encoder model is superior to the baseline model. However, the work of Wang et al. [34] did not fully utilize the information brought by the graph structure. We note that Peng et al. [55] proposed a GCN network based on bipartite graphs to predict potential carcinogenic genes, and their work shows that this network can extract low-order information brought by the graph structure well. In addition, the Deep Forest model proposed by Zhou et al. [45] outperforms traditional machine learning methods on multiple datasets. Inspired by these works, we designed a widely effective computational framework DAEGCNDF for predicting potential microbial-disease associations. The flowchart of the DAEGCNDF model is shown in Fig. 1, which can be divided into five parts: (1) Similarity calculation (Fig. 1A), (2) Similarity fusion (Fig. 1B), (3) Extraction of low-order features (Fig. 1C), (4) Extraction of high-order features (Fig. 1D), (5) Feature fusion and prediction using deep forest model (Fig. 1E).

The work of Wang et al. [34] suggests that utilizing the multiple similarities between microbes and diseases can enhance model performance. As shown in Fig. 1A, B, we calculated four types of similarities for both microbes and diseases, and integrated these similarities. To extract the information brought by the graph structure and avoid over-smoothing, as shown in Fig. 1C, we used a two-layer GCN module to extract the low-rank features of the nodes. To compensate for the inability of the GCN module to extract higher-rank information, as shown in Fig. 1D, we introduced a four-layer Auto-Encoder model to extract the high-rank features of the nodes. Finally, we concatenated the low-rank features and high-rank features, and used the deep forest model for prediction.

#### GCN module

The Graph Convolutional Model can learn the hidden layer representation of nodes by the features of neighboring nodes and local graph structure. This model requires the adjacency matrix of the graph and the feature matrix of nodes as initial inputs. Inspired by Peng et al. [55], the specific process of the GCN module is as follows: First, matrices $\textbf{FuM}$ and $\textbf{FuD}$ are used as the initial features of microbes and diseases. To make the dimensions of these two initial features consistent, we use Eq. (16) for dimension reduction. Then, we use Eq. (17) to aggregate neighborhood features. Finally, we use Eq. (18) for local graph structure learning.

16 $$\begin{aligned} \textbf{LinM}= & {} \textbf{FuM}\cdot \textbf{W}^{(0)}_{M}+b_{M},\nonumber \\ \textbf{LinD}= & {} \textbf{FuD}\cdot \textbf{W}^{(0)}_{D}+b_{D}. \end{aligned}$$

17 $$\begin{aligned} \textbf{NM}^{(1)}= & {} \tilde{\textbf{P}}\cdot \textbf{LinD} \cdot \textbf{W}^{(1)}_{1},\nonumber \\ \textbf{ND}^{(1)}= & {} \tilde{\textbf{P}}^{T}\cdot \textbf{LinM} \cdot \textbf{W}^{(1)}_{1}. \end{aligned}$$

18 $$\begin{aligned} \textbf{GM}^{(1)}= & {} \left( \left( \tilde{\textbf{P}}\cdot \textbf{LinD}\right) \odot \textbf{LinM} \right) \cdot \textbf{W}^{(1)}_{2} + b_{1},\nonumber \\ \textbf{GD}^{(1)}= & {} \left( \left( \tilde{\textbf{P}}^{T}\cdot \textbf{LinM}\right) \odot \textbf{LinM}\right) \cdot \textbf{W}^{(1)}_{2} + b_{1}. \end{aligned}$$

Where $\textbf{W}^{(0)}_{M} \in R^{1177 \times h_{1}}, \textbf{W}^{(0)}_{D} \in R^{134 \times h_{1}}, \textbf{W}^{(1)}_{1} \in R^{h_{1} \times h_{2}}, \textbf{W}^{(1)}_{2} \in R^{h_{1} \times h_{2}}$ are learnable weight matrices, while $b_{M}, b_{D}, b_{1}$ are learnable bias vectors with a dimension of $h_{1}$. $\textbf{D}_{1}=\sum _{j}\textbf{Y}_{ij}+1$ and $\textbf{D}_{2}=\sum _{i}\textbf{Y}_{ij}+1$ are diagonal matrices, $\tilde{\textbf{P}}=\textbf{D}^{-\frac{1}{2}}_{1} \textbf{Y} \textbf{D}^{-\frac{1}{2}}_{2}$. $\odot$ represents the element-wise multiplication.

After calculating according to the formula above, as shown in Eq. (19), by adding the aggregated neighborhood features and the learned local graph structure information and activating them with an activation function, we can obtain the low-rank features of nodes with neighbor node features and local graph structure information. It should be noted that Eqs. (17) and (18) constitute the first layer of the GCN module. We can summarize the process above into the following formula:

19 $$\begin{aligned} \textbf{LM}^{(1)}= & {} GCN^{(1)}_{M}\left( \{\textbf{LinM},\textbf{LinD},\textbf{Y}\}_{i \in N(M)} \right) \nonumber \\= & {} \sigma \left( \textbf{NM}^{(1)}+\textbf{GM}^{(1)}\right) ,\nonumber \\ \textbf{LD}^{(1)}= & {} GCN^{(1)}_{D}\left( \{\textbf{LinM},\textbf{LinD},\textbf{Y}\}_{i \in N(D)} \right) \nonumber \\= & {} \sigma (\textbf{ND}^{(1)}+\textbf{GD}^{(1)}). \end{aligned}$$

Where *N*(*M*) and *N*(*D*) respectively represent the set of neighbors for microbes and diseases in the network. $\sigma$ represents the ReLU activation function.

Like a general GCN, our GCN module can also stack multiple graph convolution layers. Let *l* represent the number of layers of the graph convolution layer, and $\textbf{LM}^{(l)}$ and $\textbf{LD}^{(l)}$ respectively represent the final microbial features and disease features learned by the GCN model from the microbe-disease network, that is, the low-rank features of microbes and diseases. Formally, a $l \ge 2$-layer GCN model can be represented by the following Eq. (20). In this paper, the number of layers in our GCN module is 2, that is, $l=2,\textbf{LM}=\textbf{LM}^{(l)},\textbf{LD}=\textbf{LD}^{(l)}$.

20 $$\begin{aligned} \textbf{LM}^{(l)}= & {} GCN^{(l)}_{M}\left( \{\textbf{LM}^{(l-1)},\textbf{LD}^{(l-1)},\textbf{Y}\}_{i \in N(M)} \right) ,\nonumber \\ \textbf{LD}^{(l)}= & {} GCN^{(l)}_{D}\left( \{\textbf{LM}^{(l-1)},\textbf{LD}^{(l-1)},\textbf{Y}\}_{i \in N(D)} \right) . \end{aligned}$$

As shown in Eq. (21), the association matrix $\textbf{Y}$ of microbes and diseases is reconstructed by using the inner product of the low-rank features of microbes and diseases output by the GCN model. Here, $\sigma$ represents the sigmoid activation function. In addition, we use Eq. (22) as the loss function for the reconstruction of the microbe-disease association matrix.

21 $$\begin{aligned} \hat{\textbf{Y}}= & {} \sigma \left( \textbf{LM} \cdot \textbf{LD}^{T} \right) . \end{aligned}$$

22 $$\begin{aligned} L= & {} -\frac{1}{n}\left( \sum \limits _{\langle i,j \rangle \in E} \log {\hat{y}_{ij}} + \sum \limits _{\langle i,j \rangle \in Neg} \left( 1-\log {\hat{y}_{ij}}\right) \right) . \end{aligned}$$

Where *E* represents the edge set of the microbe-disease network, while *n* is the number of edges. *Neg* refers to the set of negative samples, which is of size *n* and obtained by negative sampling, while $\hat{y}_{ij}$ represents the value of the reconstructed adjacency matrix $\hat{\textbf{Y}}$.

#### Deep auto-encoder module

Deep Auto-Encoder is an unsupervised learning model that can efficiently learn the latent information of sample data. This model typically consists of an encoder and a decoder. The aim of the deep Auto-Encoder is to reconstruct the input, thereby enabling the neural network to learn the most informative latent features of the input data, making it widely used in feature extraction.

For any disease $d_{i}$, we take the *i*-th row $\textbf{FuD}_{i}$ of matrix $\textbf{FuD}$ as its initial feature vector; similarly, for any microbe $m_{i}$, we take the *j*-th row $\textbf{FuM}_{j}$ of matrix $\textbf{FuM}$ as its initial feature vector. We concatenate $\textbf{FuD}_{i}$ and $\textbf{FuM}_{i}$ to obtain the feature vector of disease-microbe pair $d_i-m_j$, at which point the dimension of the feature vector of disease-microbe pair $d_i-m_j$ is 1311. We use a deep Auto-Encoder to extract the effective features of disease-microbe pairs. Specifically, the encoder and decoder of the model can be represented by Eqs. (23) and (24) respectively.

23 $$\begin{aligned} z^{(k)}= & {} \sigma ^{(k)}_{e}\left( \textbf{W}^{(k)}_{e}z^{(k-1)} + b^{(k)}_{e}\right) . \end{aligned}$$

24 $$\begin{aligned} x^{(t)}= & {} \sigma ^{(t)}_{d}\left( \textbf{W}^{(t)}_{d}x^{(t-1)} + b^{(t)}_{d}\right) . \end{aligned}$$

Where $k \ge 1$ and $t\ge 1$ represent the number of layers in the encoder and decoder, respectively. Following the study of Wang et al [34], we set them both to 4. $\sigma ^{(k)}_{e}$ and $\sigma ^{(t)}_{d}$ represent the activation functions of the encoder and decoder respectively, and in this paper, they are both set to sigmoid function. $\textbf{W}^{(k)}_{e}$, $b^{(k)}_{e}$ and $\textbf{W}^{(t)}_{d}$, $b^{(t)}_{d}$ are the learnable parameters of the encoder and decoder. In addition, $z^{(0)}$ is the initial input data *x*, and $x^{(0)}=z^{(4)}$.

As shown in Eq. (25), the model’s loss is composed of mean squared error and KL divergence, where $\theta$ is the weight coefficient.

25 $$\begin{aligned} L_{DAE}=MSE(x,x^{(4)}) + \theta \cdot KL(x,x^{(4)}). \end{aligned}$$

Ultimately, the $z^{(4)}$ obtained by the model is treated as the high-order feature vector of the disease-microbe pair.

#### Prediction of microbe-disease associations by deep forest model

Deep Forest is a decision tree ensemble method proposed by Zhou et al in 2018 [45]. This method first preprocesses the input features using multi-granularity scanning, then inputs the obtained feature vectors into a cascading forest for training, and uses cross-validation to generate each cascade, effectively avoiding overfitting. As shown in Fig. 1E, we take the *i*-th row $\textbf{LD}_{i}$ of the low-rank feature matrix $\textbf{LD}$ of the disease extracted by the GCN module and the *j*-th row $\textbf{LM}_{j}$ of the low-rank feature matrix $\textbf{LM}$ of the microorganism as the low-rank feature vectors of disease $d_i$ and microorganism $m_j$ respectively. By concatenating $\textbf{LD}_{i}$ and $\textbf{LM}_{j}$, we can obtain the low-rank feature vector of the disease-microorganism pair $d_i-m_j$. Afterwards, we concatenate the high-rank feature vector and the low-rank feature vector to obtain the final feature vector of the disease-microbe pair. Finally, we input the final feature vector of the disease-microbe pair into the Deep Forest model for latent microbe-disease associations prediction.

**Table 3:** The experimental results of the DAEGCNDF model based on 10-fold cross-validation

Dataset:	MDAID					
Testing set	Acc(%)	Pre (%)	Recall (%)	F1-score (%)	AUC (%)	AUPR (%)
1	92.34	92.35	92.33	92.34	97.71	98.01
2	90.68	90.62	90.68	90.65	97.42	97.12
3	90.01	90.03	90.01	90.01	96.65	96.37
4	89.90	89.89	89.86	89.87	96.04	95.65
5	91.01	90.97	91.05	91.00	97.42	97.61
6	91.79	91.79	91.79	91.79	97.55	97.71
7	90.12	90.11	90.19	90.12	95.87	95.36
8	89.67	89.65	89.69	89.66	96.36	96.40
9	90.22	90.34	90.13	90.19	97.03	96.87
10	93.11	93.14	93.10	93.11	97.90	97.90
Average	90.89 ± 1.16	90.89 ± 1.17	90.88 ± 1.16	90.87 ± 1.17	97.00 ± 0.72	96.90 ± 0.94

Dataset:	HMDAD					
Testing set	Acc(%)	Pre (%)	Recall (%)	F1-score (%)	AUC (%)	AUPR (%)
1	91.11	91.25	90.84	91.00	97.66	97.65
2	90.00	90.00	90.18	89.89	97.07	9755
3	85.56	86.25	85.87	85.54	94.85	94.86
4	86.67	86.84	86.84	86.67	96.14	96.28
5	86.67	87.16	86.94	86.67	95.45	96.30
6	83.33	83.90	83.78	83.33	93.25	94.78
7	92.22	92.16	92.26	92.20	97.17	97.49
8	90.00	90.18	90.00	90.00	97.93	98.11
9	88.89	89.00	88.69	88.80	96.78	96.82
10	86.67	86.74	86.67	86.66	96.94	97.37
Average	88.11 ± 2.77	88.35 ± 2.58	88.21 ± 2.62	88.08 ± 2.75	96.32 ± 1.44	96.72 ± 1.16

## Result

### Parameter details and model evaluation

We implemented our model using PyTorch and PyG, with both the GCN module and the Deep Auto-Encoder module utilizing Adam as the optimizer. For the GCN module, we set the number of network layers to 2, with the dimensions of the hidden layer and output layer set to 256 and 128 respectively. We used a default dropout rate of 0.5, and set the number of model training iterations and learning rate to 1000 and 0.001 respectively. For the Deep Auto-Encoder module, as previously mentioned, we set the number of layers for both the encoder and decoder to 4, with the dimensions of each network layer being 1311, 1152, 576, 288, 144, 288, 576, 1152, and 1131 respectively (see Fig. 1E). The number of model training iterations and initial learning rate were set to 150 and 0.01 respectively, with ReduceLROnPlateau used for automatic optimization of the learning rate. For the Deep Forest model, we set ’n_estimators’ and ’criterion’ to 17 and ’entropy’, respectively.

In this study, we conducted experiments using 10-fold cross-validation and evaluated the model using a variety of metrics, namely AUC, AUPR, Recall, Precision (Pre), Accuracy (Acc), and F1-score. Considering that MDAID is a large dataset, to further demonstrate the performance of our model, we also conducted experiments on the HMDAD dataset. As indicated in Table 3, our model achieved good performance on both datasets.

**Table 4:** Comparison of methods for selecting negative samples based on MDAID dataset

Methods	AUC (%)	AUPR (%)
Random sampling	92.88	92.85
KMeans clustering	**97.00**	**96.90**
Gaussian mixture clustering	96.95	96.85
Spectral coclustering	93.71	93.98
Spectral biclustering	94.93	94.96

The bold result indicates the best one in each column

### Comparison of methods for selecting negative samples

We noticed that in the microbe-disease association matrix $\textbf{Y}$, a value of “1” indicates the presence of a microbe-disease association, indicating a positive sample. Conversely, a value of “0” represents an unknown or negative sample. This suggests that there is an issue with false negatives in these negative samples, highlighting the importance of selecting reliable negative samples during the model training phase. Wang et al. [34] and Peng et al. [33] employed KMeans clustering to group negative samples into 23 categories and subsequently randomly selected 196 negative samples from each category, resulting in a total of 4508 negative training samples. The advantage of this approach lies in ensuring that negative samples contribute to model training for each type of data feature, thereby avoiding biased learning during model training. In this study, we employ five methods for selecting negative samples: random sampling, KMeans clustering sampling, Gaussian mixture clustering sampling, spectral co-clustering sampling, and spectral bi-clustering sampling.

As shown in Table 4, sampling negative samples by clustering methods can effectively improve model performance. Among them, KMeans clustering sampling has the best effect on improving model performance, improving model performance by about 4$\%$ compared to random sampling. However, the effect of Gaussian mixture clustering sampling on improving model performance is almost the same as that of KMeans clustering sampling.

**Table 5:** Results of the ablation experiments on model DAEGCNDF based on 10-fold cross-validation

Dataset:	MDAID					
Experiments	Acc(%)	Pre (%)	Recall (%)	F1-score (%)	AUC (%)	AUPR (%)
LRF	90.75	90.75	90.74	90.74	96.85	96.78
HRF	86.67	86.67	86.65	86.65	94.63	94.64
LHRF	**90.89**	**90.89**	**90.88**	**90.87**	**97.00**	**96.90**

Dataset:	HMDAD					
Experiments	Acc(%)	Pre (%)	Recall (%)	F1-score (%)	AUC (%)	AUPR (%)
LRF	85.56	85.86	85.77	85.56	95.89	96.11
HRF	85.89	85.48	85.56	85.51	95.04	96.12
LHRF	**88.11**	**88.35**	**88.21**	**88.08**	**96.32**	**96.72**

The bold result indicates the best one in each column

### Ablation experiments

To evaluate the impact of low-rank and high-rank features on the predictive performance of the model, we divided the features of the disease-microbe pairs into three groups: LRF, HRF, and LHRF. Group LRF represents predictions made using only low-rank features, Group HRF represents predictions made using only high-rank features, and Group LHRF represents predictions made after concatenating low-rank and high-rank features.

From Table 5, we can see that the low-rank features of disease-microorganism pairs contribute more to the model performance than the high-rank features. This may be due to our GCN module’s ability to effectively aggregate the features of diseases and microorganisms through neighboring nodes. Furthermore, when low-rank and high-rank features are combined, the model’s performance surpasses that of predictions made using only a single feature.

**Table 6:** Experimental results of different classifiers based on 10-fold cross-validation

Dataset:	MDAID					
Experiments	Acc(%)	Pre (%)	Recall (%)	F1-score (%)	AUC (%)	AUPR (%)
MLP	90.25	90.24	90.26	90.24	96.54	96.30
LR	85.10	85.10	85.09	85.08	92.87	92.05
SVM	89.67	89.67	89.65	89.654	95.54	94.84
NB	79.47	79.75	79.47	79.40	85.66	86.89
DT	85.49	85.48	85.48	85.47	90.04	91.22
ABC	84.61	84.61	84.59	84.59	92.91	92.28
GBC	88.53	88.55	88.53	88.51	95.45	94.99
KNN	87.51	87.67	87.52	87.48	94.01	94.23
RF	90.38	90.38	90.39	90.37	96.68	96.48
DF	**90.89**	**90.89**	**90.88**	**90.87**	**97.00**	**96.90**

Dataset:	HMDAD					
Experiments	Acc(%)	Pre (%)	Recall (%)	F1-score (%)	AUC (%)	AUPR (%)
MLP	**88.11**	88.20	88.14	88.08	95.48	95.87
LR	86.67	83.93	83.71	83.59	89.61	84.37
SVM	87.78	88.19	87.94	87.73	94.27	91.89
NB	77.44	78.11	77.46	77.30	84.48	85.82
DT	84.00	84.28	84.01	83.94	89.40	90.92
ABC	85.33	85.65	85.43	85.28	93.47	91.96
GBC	86.44	86.61	86.50	86.40	95.45	95.87
KNN	87.00	87.22	87.03	86.95	93.83	94.34
RF	87.44	87.63	87.47	87.41	95.41	95.86
DF	**88.11**	**88.35**	**88.21**	**88.08**	**96.32**	**96.72**

The bold result indicates the best one in each column

**Table 7:** The experimental results of different models based on 10-fold cross-validation

Dataset: MDAID	Dataset: HMDAD			
Methods	AUC (%)	AUPR (%)	AUC (%)	AUPR (%)
NTSHMDA [56]	75.67	18.56	74.97	18.19
NCPHMDA [57]	79.89	17.86	79.01	17.43
LRLSHMDA [58]	79.92	18.19	79.99	18.21
KATZHMDA [25]	81.35	19.78	81.44	19.89
ABHMDA [33]	94.78	92.89	94.11	94.61
KGNMDA [40]	93.87	94.07	93.15	94.13
DSAE_RF [34]	94.48	94.31	94.49	94.69
DAEGCNDF(our)	**97.00**	**96.90**	**96.32**	**96.71**

The bold result indicates the best one in each column

### Comparison of different classifiers

To evaluate the contribution of Deep Forest (DF) to predictive performance, we selected nine benchmark models, including a three-layers MLP neural network commonly used as a benchmark model, and eight traditional machine learning models. These are Logistic Regression (LR), Support Vector Machine (SVM), Naive Bayes (NB), Decision Tree (DT), AdaBoost Classifier (ABC), Gradient Boosting Classifier (GBC), K-Nearest Neighbors (KNN), and Random Forest(RF). The prediction results are shown in Table 1.

As can be seen from the results in Table 6, the Deep Forest classifier outperforms the other nine benchmark classifiers across all evaluation metrics. Furthermore, these results indicate that while Random Forest outperforms other traditional machine learning models, Deep Forest, as an improved model of Random Forest, demonstrates superior performance. Therefore, our choice of Deep Forest as the final classifier is both reasonable and reliable.

### Comparison of other methods

To further evaluate the performance of our model, we selected six of the latest microbe-disease associations prediction methods for comparison with our model, based on the dataset in this paper and 10-fold cross-validation. The names of the models and the experimental results are shown in Table 7.

From the experimental results in Table 7, it is evident that our model, DAEGCNDF, outperforms the benchmark models in terms of AUC and AUPR values. Specifically, our model achieved an AUC value of $97.00\%$ and an AUPR value of $96.90\%$, which are approximately $2.22\%$ and $2.59\%$ higher than the second-place model, respectively. We attribute the optimal performance of our DAEGCNDF model to four main reasons. Firstly, the GCN module employed in our model effectively captures low-order features from bipartite graphs representing microbes and diseases with a graph structure. Secondly, the DAE module successfully extracts complex high-rank features from disease-microbe pairs, thereby eliminating noise present in these initial features after undergoing DAE processing. Furthermore, by combining both low-rank and high-rank features, we are able to better represent information pertaining to disease-microbe pairs and consequently enhance classifier performance. Lastly, the deep forest cascade structure utilized by our model enables effective utilization of input features for prediction purposes.

## Case studies

To evaluate the performance of DAEGCNDF further, we conducted two types of case studies on this model: predicting potential microbe-disease associations based on known information and predicting new microbe-disease associations based on unknown information. In the first type of case study, all known microbe-disease association information was used for training purposes. Subsequently, predictions were made for all unknown associations corresponding to a given disease while ranking them according to their prediction scores. Finally,the top ten microbes with highest scores were validated using literature sources. In the second type of case study, the disease under study was treated as a completely new disease, and its association information with microbes would be removed before model training, which means that there is no information about this disease during model training. Similar to the first type of case study, we ranked the scores of all microbes corresponding to the same disease and took the top 10 microbes for validation by relevant literature. It is important to note that conducting the second type of case study allows us to assess our model’s ability to predict microbial associations with new diseases when no prior disease-microbe related information is available.This reflects how well our model can guide actual experiments.

Colorectal cancer is a common malignant tumor in the gastrointestinal tract, with early symptoms often not obvious [59]. Therefore, about 20$\%$ of newly diagnosed colorectal cancer patients have already experienced cancer cell metastasis [60]. Early diagnosis of colorectal cancer is of great significance for the treatment of the disease and improving the survival time of patients [61]. Although the cause of its onset is not yet fully understood, more and more evidence suggests that gut microbes have an impact on the occurrence, progression, metastasis, treatment, and prognosis of colorectal cancer. For example, Gao et al. [62] found that Lactococcus and Fusobacterium are relatively enriched in colorectal cancer tissues. Wang et al. [63] found that Salmonella enterica is involved in the progression of colorectal cancer. Therefore, further study of the relationship between colorectal cancer and microbes will help us further understand its pathogenesis and is of great significance for its early screening, auxiliary diagnosis, and assistance. In view of this, we chose colorectal cancer for the two types of case studies above. As can be seen from Table 8, in the first type of case study, 8 out of the top 10 microbes predicted to be associated with colorectal cancer were confirmed by literature. In addition, in the second type of case study (see Table 9), all of the top 10 microbes predicted to be associated with colorectal cancer were confirmed by literature.

Autoimmune hepatitis is a chronic progressive inflammatory disease of the liver mediated by autoimmune reactions, which can manifest in acute or chronic forms [64, 65]. In severe cases, it can rapidly progress to cirrhosis and liver failure, threatening life [66]. The disease occurs worldwide, with an incidence rate exceeding forty-two per hundred thousand in certain ethnic groups [67]. The disease requires timely and long-term treatment, and untimely or improper treatment can greatly affect the patient’s 10-year survival rate [68]. Currently, a large amount of research has confirmed that autoimmune hepatitis is related to changes in the composition of the gut microbiota. For example, Liwinski et al. [69] found that Bifidobacterium affects the remission of autoimmune hepatitis. Wei et al. [70] found that Veillonella not only has a strong correlation with autoimmune hepatitis but also affects the progression of hepatitis. Lou et al. [71] found that a combination of Bacteroides, Ruminococcaceae, Lachnospiraceae, Veillonella, Roseburia, and Ruminococcaceae can distinguish autoimmune hepatitis patients from healthy controls, suggesting that certain microbes or their combinations can serve as markers for autoimmune hepatitis. Therefore, it is practically significant to choose autoimmune hepatitis as a case study. Tables 10 and 11 reveal that, of the top 10 microbes projected to potentially associate with autoimmune hepatitis, 8 have been validated by literature. Furthermore, among the top 10 microbes predicted to form new associations with autoimmune hepatitis, five have been substantiated by literature.

Examining the four experimental outcomes from the aforementioned pair of case studies, our model exhibits strong performance across both types of experiments. This demonstrates the model’s robust practical guidance capabilities. Consequently, our model’s predictive results can be leveraged to enhance the efficiency of traditional biomedical experiments and reduce their duration.

**Table 8:** Predicting the top 10 potential microbes associated with colorectal cancer by DAEGCNDF

Colorectal cancer		
Rank	Microbes	Evidence
1	Veillonella	PMID: 22761885
2	Clostridium	PMID: 26992426
3	Sporobacter	Unconfirmed
4	Ruminococcus gnavus	PMID: 36893736
5	Corynebacterium	PMID: 27863401
6	Vivictivallis	Unconfirmed
7	Holdemania	PMID: 23733170
8	Oscillospira	PMID: 31358825
9	Subdoligranulum	PMID: 29995183
10	Shigella	PMID: 35663463

**Table 9:** Predicting the top 10 new microbes associated with colorectal cancer by DAEGCNDF

Colorectal cancer		
Rank	Microbes	Evidence
1	Lactobacillus	PMID: 15828052
2	Lachnospiraceae	PMID: 28988196
3	Prevotella	PMID: 33488574
4	Streptococcus	PMID: 21247505
5	Ruminococcus	PMID: 36585646
6	Pseudomonas	PMID: 25699023
7	Megasphaera	PMID: 35727391
8	Fusobacterium	PMID: 25699023
9	Enterobacteriaceae	PMID: 25182170
10	Porphyromonas	PMID: 33425779

**Table 10:** Predicting the top 10 potential microbes associated with autoimmune hepatitis(AIH) by DAEGCNDF

AIH		
Rank	Microbes	Evidence
1	Prevotella	PMID: 32640728
2	Lachnospiraceae	PMID: 32850468
3	Faecalibacterium	PMID: 32383181
4	Bacteroides	PMID: 32850468
5	Roseburia	PMID: 32850468
6	Actinomyces	PMID: 34094998
7	Dialister	PMID: 32640728
8	Rothia	Unconfirmed
9	Ruminococcus	PMID: 36519162
10	Faecalibacterium prausnitzii	Unconfirmed

**Table 11:** Predicting the top 10 new microbes associated with autoimmune hepatitis(AIH) by DAEGCNDF

AIH		
Rank	Microbes	Evidence
1	Ruminococcus	PMID: 36519162
2	Corynebacterium	Unconfirmed
3	Acinetobacter	Unconfirmed
4	Lactobacillus	PMID: 26191211
5	Pseudomonas	Unconfirmed
6	Bacteroides	PMID: 32850468
7	Firmicutes	Unconfirmed
8	Fusobacterium	PMID: 29969462
9	Parabacteroides	PMID: 32640728
10	Roseburia	PMID: 32850468

## Discussion and conclusion

The human body is a vast ecosystem teeming with microbes, many of which play a pivotal role in our health and the onset, progression, and treatment of diseases. As such, understanding the intricate relationships between these microbes and diseases is crucial for disease prevention, clinical practice, and biomedical research. Traditional biomedical experiments in this field often face hurdles due to their lengthy duration, high costs, and strict requirements for experimental conditions. While computational methods offer a way to circumvent these challenges to some degree. They are not without their own limitations. These include the inadequate extraction and utilization of data features, less-than-optimal methods for selecting reliable negative samples, and a lack of precision in model predictions.

In this study, we introduce DAEGCNDF, a novel computational model designed to predict associations between microbes and diseases. Our approach involves calculating four distinct types of similarity for both microbes and diseases, which are then fused to generate a comprehensive set of initial features. We employ GCN to extract high-rank features of diseases and microbes, while the DAE module is used to distill low-rank features of disease-microbe pairs. In the process of selecting negative samples for training, we compared five different sampling methods to ensure the selection of reliable negative samples. Our findings indicate that KMeans clustering sampling and Gaussian mixture cluster clustering sampling enhance model performance by approximately 4$\%$. In the final step, we concatenate the low and high-rank features of disease-microbe pairs and utilize a deep forest for predicting potential microbe-disease associations. Through ablation experiments, classifier selection experiments, and case studies, our computational framework demonstrates significant potential in identifying potential microbe-disease associations.

From the experimental results, the performance of our model is superior to the baseline model, and we believe there are four main reasons. First, the GCN variant module suitable for bipartite graphs can effectively extract the low-order information of nodes. Second, the DAE module can effectively extract the high-order features of the microbe-disease pair. Third, unlike the traditional random selection of negative samples, we used KMean for negative sample sampling. Fourth, the performance of the deep forest classification is superior to traditional machine learning methods.

Nonetheless, our model does have certain limitations that warrant further refinement in the future. This includes the need to devise superior methods for selecting reliable negative samples and to delve into the mathematical principles that underpin the differences in these methods. Moreover, the interplay between drugs, ncRNA, microbes, and diseases presents an opportunity for extracting novel features of microbes and diseases. This is an area that is yet to be fully explored. Our future work will concentrate on these two pivotal aspects.

## Data Availability

The datasets and corresponding codes are available at https://github.com/cuntjx/microbe.
